# Synthetic biology routes to bio-artificial intelligence

**DOI:** 10.1042/EBC20160014

**Published:** 2016-11-30

**Authors:** Darren N. Nesbeth, Alexey Zaikin, Yasushi Saka, M. Carmen Romano, Claudiu V. Giuraniuc, Oleg Kanakov, Tetyana Laptyeva

**Affiliations:** ^1^ Department of Biochemical Engineering, University College London, Bernard Katz Building, London WC1E 6BT, U.K.; ^2^ Department of Mathematics, University College London, Gower Street, London WC1E 6BT, U.K.; ^3^ Institute for Women's Health, University College London, London WC1E 6AU, U.K.; ^4^ School of Medicine, Medical Sciences and Nutrition, Institute of Medical Sciences, University of Aberdeen, Foresterhill, Aberdeen AB25 2ZD, U.K.; ^5^ Department of Physics, Institute for Complex Systems and Mathematical Biology, Meston Building, Old Aberdeen, Aberdeen, U.K.; ^6^ Oscillation Theory Department, Lobachevsky State University of Nizhniy Novgorod, Novgorod, Russia; ^7^ Department of Control Theory and Systems Dynamics, Lobachevsky State University of Nizhniy Novgorod, Novgorod, Russia

**Keywords:** artificial intelligence, gene networks, synthetic biological circuits, synthetic biology

## Abstract

The design of synthetic gene networks (SGNs) has advanced to the extent that novel genetic circuits are now being tested for their ability to recapitulate archetypal learning behaviours first defined in the fields of machine and animal learning. Here, we discuss the biological implementation of a perceptron algorithm for linear classification of input data. An expansion of this biological design that encompasses cellular ‘teachers’ and ‘students’ is also examined. We also discuss implementation of Pavlovian associative learning using SGNs and present an example of such a scheme and *in silico* simulation of its performance. In addition to designed SGNs, we also consider the option to establish conditions in which a population of SGNs can evolve diversity in order to better contend with complex input data. Finally, we compare recent ethical concerns in the field of artificial intelligence (AI) and the future challenges raised by bio-artificial intelligence (BI).

## Introduction

Artificial intelligence (AI) can be defined as the decision-making capabilities of machines [1]. Machines are most commonly regarded as designed, multi-part objects that perform predetermined mechanical tasks. To date all commercial machines are constructed using electronics directed by sets of instructions (algorithms) encoded within circuits patterned onto semiconductor materials such as silicon. Machine learning can occur when the algorithms that control the machine are written such that the algorithms themselves can independently use prior data sets to inform future decisions.

Human learning, by contrast, is understood to be a phenomenon that emerges in part from the dynamic and adaptive exchange of information between neurons in the brain and within individual neuronal cells. Individual cells can adapt to and anticipate environmental signals, such as the onset of stress and the availability of nutrients. For example, cells of the mammalian immune system can acquire memory of previous pathogen invasions and prepare for future infections. In experiments, the slime mould *Physarum polycephalum*, a single-celled organism, found the shortest path between two points in a labyrinth [2,3], and anticipated future events that it had previously experienced on a periodic basis [4].

Networks of genes [5] and enzymes [6] have been described in terms of their ability to support adaptive behaviours. However, research is ongoing into the network topologies and behaviours that underlie single cell learning. A key question is whether learning in single cells occurs in a manner analogous to multicellular systems, or with an architecture that is predetermined by genetically encoded programs. The development of synthetic biology in recent years provides a novel avenue to address this question from a biological engineering perspective.

Association and classification of external stimuli are two fundamental concepts used to define learning in the field of AI. A number of theoretical studies indicate that single cells can exhibit these types of learning [7–9]. Cell-free biological systems have also been established which exploit DNA strand hybridisation and displacement to perform neural network computations [10]. To date, however, no artificial single-cell-based learning system has been realised experimentally. Rapid development of synthetic biology in the previous decade has now made engineering of such a system feasible.

In this review, we discuss a selection of synthetic biologists’ efforts to design, model, build and test synthetic gene networks (SGNs) that enable living cells to associate and classify external stimuli. In doing so we hope to stimulate researchers to consider and debate how synthetic biology could be used to implement AI using biological material as an alternative to the silicon, metal and plastic materials used in conventional AI.

While mathematical models were applied in the development and analysis of the SGNs discussed here, this review focuses on the biological aspects of SGN. As such, a complete description of the relevant models is not necessary to understand the concepts presented here. Readers who do wish to examine the mathematical models further should refer to the cited literature and reviews by Bates et al. [11] and Borg et al. [12]. Specific technical details can be provided via the corresponding author.

### Supervised learning in artificial intelligence: students, teachers and classification

**Figure 1:**
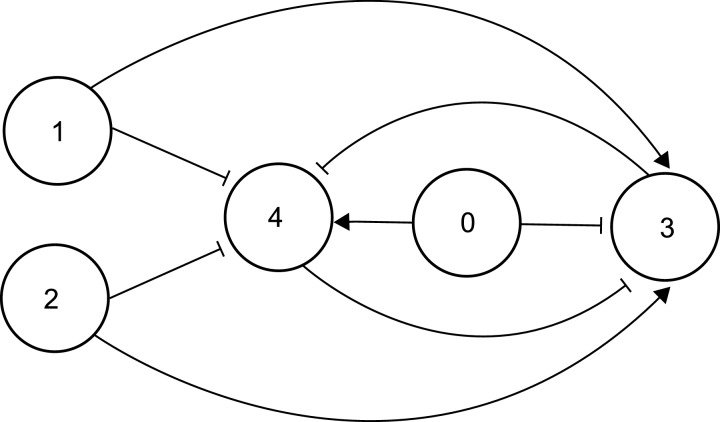
A synthetic gene network for linear classification A linear classifier phenotype can be achieved with a SGN comprising five nodes, depicted in the diagram as circles labelled 0, 1, 2, 3 and 4. Arrowhead connectors indicate activation of one node by another, hammerhead connectors indicate inhibition. Nodes 3 and 4 represent a toggle switch, which can flip between the state of ‘3 ON, 4 OFF’ and the state of ‘3 OFF, 4 ON’. Nodes 3 and 4 repress each other. Node 0 favours the ‘4 ON’ state and inhibits the ‘3 ON’ state. Nodes 1 and 2 represent inputs that favour ‘3 ON’ and inhibit ‘4 ON’. The output position of the 3/4 toggle switch is tipped toward ‘3 ON’ or ‘4 ON’ depending on the net activity level of nodes 1 and 2. In effect the 3/4 toggle switch classifies inputs 1 and 2. Node 0 can be used to tip the equilibrium of the toggle switch toward ‘3 ON’. This impacts how the output position of the toggle switch is influenced by nodes 1 and 2. In this way, the weighting of the classification threshold can be set by the activity of node 0. This scheme is proposed here by A.Z.

A key goal of machine learning is the development of algorithms that can infer a set of rules from a predetermined ‘training’ data set. Once the training data have been analysed, the algorithm should ideally be able to correctly sort previously unseen data sets into correct categories [13] in what is termed ‘supervised learning’. One mode of this sorting, also known as ‘classification’, is to classify all data inputs into one of two states–for instance being above or below a given linear threshold. This type of supervised learning is known as linear classification and a number of algorithms have been developed to achieve this task. The perceptron is one of the earliest linear classification algorithms and has been used to identify translation initiation sites in *Escherichia coli* mRNA molecules [14]. In a perceptron algorithm, a given input signal is classed as being above or below a line (or threshold). The position of this threshold is altered as part of the learning process until all data points have been successfully classified as being above or below a linear threshold. Figure 1 sets out a scheme for biological implementation of a perceptron in which a toggle switch (Figure 3A) classifies the sum of two input signals being one side or another of a given threshold, resulting in expression of either RFP or GFP. The position of the threshold is determined by a central element, ‘node 0’. The nodes in this context represent one or more genes that function to repress or stimulate other nodes.

### Supervised learning in synthetic biology: student cells and teacher cells

**Figure 2:**
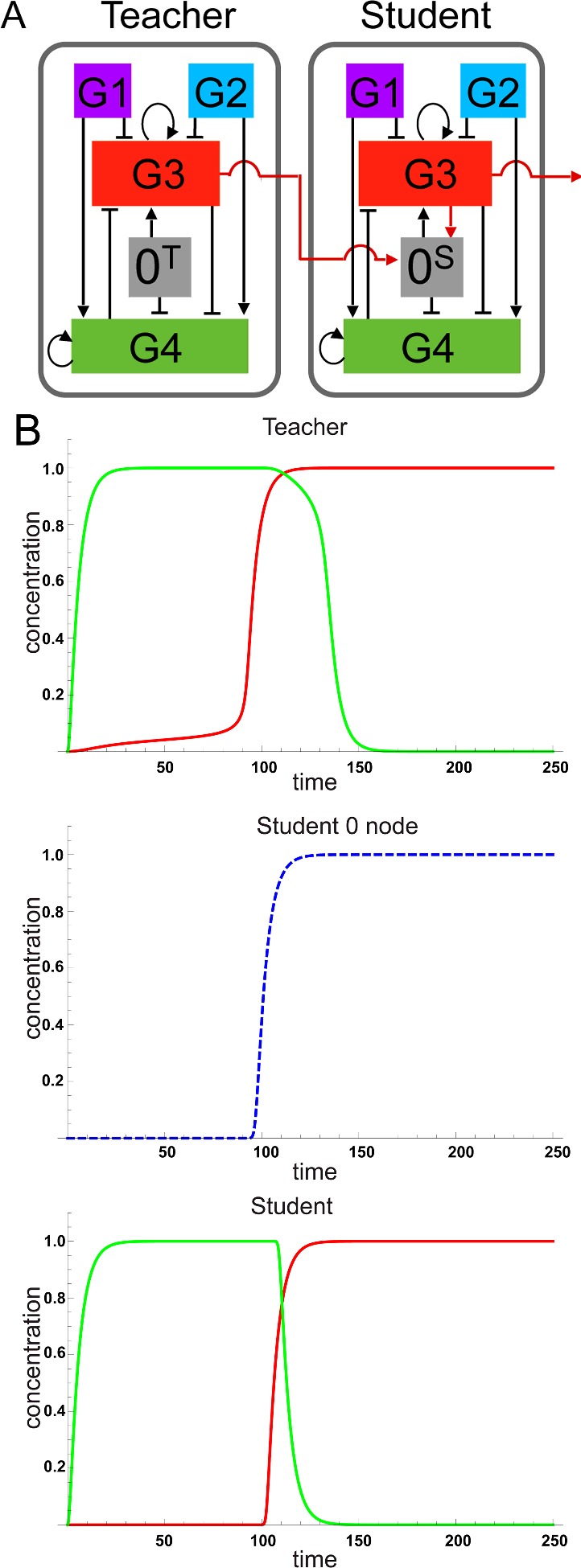
Linear classification with a biological student–teacher network (**A**) Teacher and student cells both contain SGNs encoding the five nodes described in Figure 1, but labelled here as 0, G1, G2, G3 and G4. Node 0 for a teacher cell is labelled 0^T^ and node 0 for a student cell is labelled 0^S^. As in Figure 1, nodes G3 and G4 comprise a toggle switch. The output position of the toggle switch is tipped toward G3, resulting in RFP expression or G4, resulting in GFP expression, depending on the net activity level of nodes G1 and G2. In effect the G3/G4 toggle switch classifies the activities of the G1 and G2 nodes as inputs. As in Figure 1, node 0 (0^T^ or 0^S^) pushes the equilibrium of the toggle switch toward G3. Unlike in Figure 1, in this BST network, activity of 0^T^ can be controlled exogenously by addition of a small molecule inducer to the growth medium. Furthermore, in addition to RFP, node G3 also directs expression of a small molecule that can traverse cell membranes and activate node 0^S^. This has the effect that, when teacher cells are in excess, the activity of 0^S^ in student cells is set (‘learned’) by the level of signal produced by teacher cells. Arrowhead connectors indicate activation of one node by another and hammerhead connectors indicate inhibition. Curled arrowhead connectors indicate auto-induction. (**B**) Mathematical simulation of the BST network learning dynamics. Outputs of the student cells: red for RFP from G3, green for GFP from G4, are constantly ‘learned’ from changes in the teacher cells which determine the activity (threshold) of node 0^S^ in the student cells. This scheme is proposed here by A.Z. and D.N. and the simulation was performed by C.G. and Y.S.

Algorithms and mathematical models for perceptron-based supervised learning can encompass a ‘teacher’ element that provides data sets and determines responses to those data, and a ‘student’ element, whose learning is directed by the teacher [15]. The biological student–teacher (BST) network consists of sets of genes within teacher and student cells that interact via promoting or repressing outputs. Taken individually, each network can be considered as a switch, with either RFP or GFP output as an indirect response to levels of a small molecule that can traverse cell membranes (Figure 2A). The classification threshold of the teacher can be adjusted externally by designing the O^T^ node to be influenced by an inducer molecule such as isopropyl β-D-1-thiogalactopyranoside (IPTG). The O^S^ node classification threshold in student cells would be set by the level of a second small molecule inducer, not IPTG, the concentration of which is influenced most strongly by teacher cells. In this way, students are effectively ‘taught’ the position of the classification threshold to use by the teachers.

Within industrial biotechnology this supervised learning could be used to optimise performance of a biotransformation step. For instance, in nature, material such as agricultural waste often consists of a diversity of substances that, collectively, are most commonly decomposed by consortia of different microbial species [16]. In conventional biotechnology, a lone species, typically *E. coli*, is engineered to express recombinant enzymes encoded by transgenes controlled by exogenous, strong, constitutive promoters, IPTG-inducible promoters or promoters present in a locus ported *en bloc* from another species. In future, synthetic consortia of different cell types could be designed in which a particular objective, or master instruction, would be set by controlling the classification threshold of teacher cells. Subsequent delivery of classification weighting instructions to different student cell types would be influenced by the biological status of teacher cells, providing a more dynamic and sensitive signalling. This particularly comes into play when consortia grow as 3D structures such as biofilms [17].

### Mathematical modelling of a biological student–teacher network

Suzuki et al. [18] proposed a mathematical model of a network using ordinary differential equations that can be applied to the network proposed in Figure 2A. The model incorporated the ability to vary the levels of gene transcription ‘noise’ (unexplained variation) used in simulations of gene network behaviour. Solving the equations of the model numerically, using a range of biologically relevant parameter levels for factors such as transcriptional noise, demonstrated the sensitivity of the BST SGN when changing the threshold of the switch within the teacher (Figure 2B). The simulation also showed that a change in the teacher is followed by a change in the student after a short delay. However, comparison with experimental observations is necessary to robustly assess the validity of this simulation.

## Associative learning

Association of two stimuli is perhaps most intuitively illustrated by the classic experiments of Pavlov [19], in which a dog learned to associate the ringing of a bell with feeding time. After simultaneous application of both stimuli, the dog learned to associate them, exhibiting the same response (salivation) to either of the two stimuli alone. Such classical associative learning is advantageous because it enables an organism to anticipate and adapt to environmental changes quickly and has been observed in all animals with bilateral symmetry so far studied [20].

### Building an associative perceptron with synthetic gene networks

**Figure 3:**
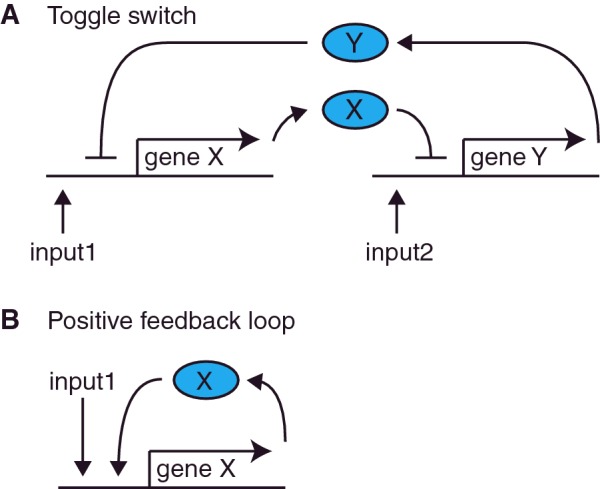
Genetic memory circuits (**A**) Genetic toggle switch. A sufficiently strong pulse of input 1 will overcome inhibition of expression of gene X caused by protein Y (Y in blue oval). Uninhibited expression of gene X will then continue as protein X (X in blue oval) also acts to inhibit expression of gene Y. Subsequently, the network can be flipped to the opposite position by a sufficiently strong pulse of input 2, which will overcome inhibition of expression of gene Y caused by protein X. Uninhibited expression of gene Y will then continue as protein Y also acts to inhibit expression of gene X. (**B**) Positive feedback loop circuit. Input 1 initiates expression of gene X. The resultant protein X then also induces express of gene X for sustained activity of the gene that will persist after the initial input 1 has ceased. Positive and negative regulations are indicated by arrows and hammerheads, respectively. These schemes have been proposed by several groups.

To perform learning tasks, cells must ‘remember’ past stimuli, and genetically encode the memory. Basic synthetic genetic memory circuits that achieve this task have been demonstrated previously, such as the genetic toggle switch depicted in Figure 3A [21] and the transcriptional positive feedback loop in Figure 3B [22]. Both of these circuits have two stable memory states dictated by the expression of the genes in the circuits.

In the genetic toggle switch (Figure 3A), either gene X or gene Y is switched on due to their mutual repression. These two memory states can be flip-flopped by two different input signals. In the positive feedback circuit (Figure 3B), the expression of gene X is switched on by an input stimulus. Once activated, the ON state is self-sustaining due to positive feedback. SGNs for associative learning can be built based upon these memory circuits. Several groups have proposed such SGNs, including Lu et al. [23] who put forward an associative learning SGN based on a toggle switch.

Elegant systems in which memory states are defined within DNA sequences have also been demonstrated by Farzadfard and Lu [24] and Yang et al. [25], using recombinase-mediated flipping of segments of genomic DNA. These systems represent potentially powerful basic research tools for discovering the provenance of different cell types. For instance, determining the events experienced by a given cell type as it matures from stem cell to terminally differentiated cell.

**Figure 4:**
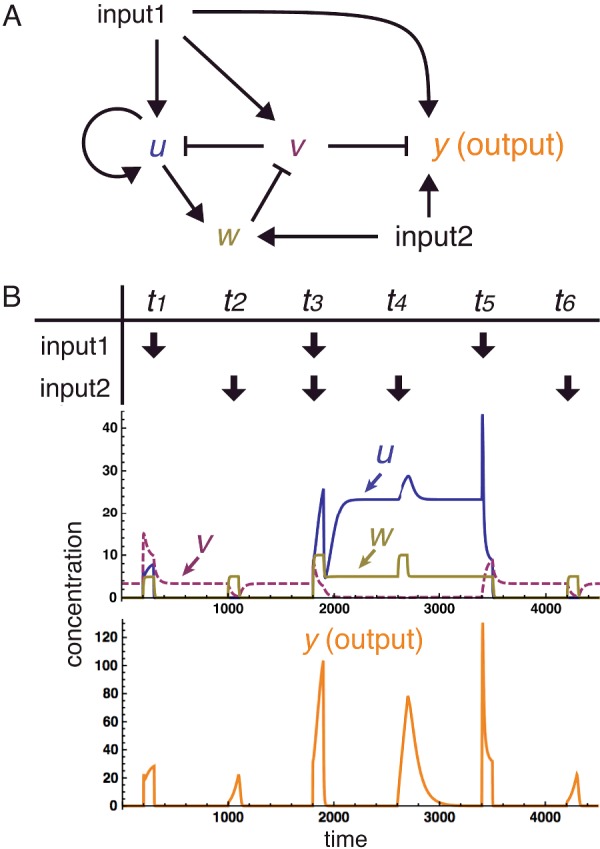
A synthetic gene network for associative learning (**A**) Schematic diagram of the PFNM associative learning network. Positive and negative regulations are indicated by arrows and hammerheads, respectively. Input 1 stimulates nodes u, v and y. Input 2 stimulates nodes w and y. (**B**) Simulation of the behaviour of the network. Either input 1 or 2 alone leads to a weak activation of the output y, at times t1 and t2. When both inputs 1 and 2 are applied simultaneously, a ‘memory’ is formed by a self-sustained expression of u due to its positive auto-regulation. Because of this memory a subsequent input 1 or input 2 alone can cause a strong induction of y. In this way the network has learned to associate inputs 1 and 2. This memory can be erased by a sufficiently large input 1 (due to the direct activation of v), bringing the system back to the default state. This scheme is proposed here by Y.S. and M.C.R. and the simulation was performed by Y.S.

For dynamic and rapid memory establishment and erasure, SGNs have been designed to be capable of associating two different environmental signals in a manner analogous to the animal learning behaviour revealed by Pavlov. One such SGN is based on the combination of a positive feedback loop memory circuit and a negative modifier (Figure 4). This ‘positive feedback/negative modified’ (PFNM) network has the important advantage that it requires only a transient signal to form a sustained memory.

Memory erasure in the PFNM circuit would be achieved post-translationally via inducible protein degradation, using a system such as the auxin-inducible protein degradation [26,27]. Steps that are achieved post-translationally allow greater network responsiveness compared with steps that are mediated by transcriptional repression. The proposed network could be implemented experimentally using genetic tools that conform to the BioBrick™ synthetic biology standard, including a transcription activator, transcription repressor, fluorescent reporter protein and a small molecule regulator of protein degradation. A mathematical model, which applies four ordinary differential equations, activating and inhibiting Hill functions and mass-action law, can be used to assess the capacity of the PFNM circuit for associative learning. Simulation using this model predicted an initial low level of network response to pulses of either input 1 or input 2 when experienced separately (Figure 4A). The network was then subjected to a pulse of both input 1 and input 2 at the same time. After this double-input pulse had been detected, the network was then predicted to give a boosted level of response to separate pulses of either input 1 or input 2 (Figure 4B). In this way, the double-input pulse establishes a memory. This memory informs an increased level of response to single inputs relative to the level of response prior to when the memory was established.

## Classification of complex inputs

**Figure 5:**
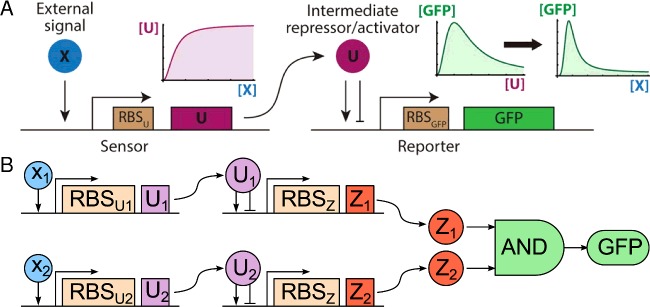
SGNs to classify input data that are not linearly separable (**A**) Sensing and response functionalities are split into separate modules. In the first module (sensor), an inducible promoter drives the expression of the transcription factor U in response to the concentration of a biological input X, such as a solute or signalling molecule. Above a certain level of X, the expression of U reaches a maximum and does not increase or decrease. In the second module (reporter), another inducible promoter drives the expression of a reporter (GFP) in response to induction by U. The promoter is activated by intermediate concentrations of U and inhibited by high concentrations of U. Thus, the resulting response function of the entire two-promoter circuit to the concentration of signalling molecule is bell shaped for the relevant values of the input signal. (**B**) In the case of two input ranges, X_1_ and X_2_, the sensor/output modules feed into an AND gate which sums the output signals as either the presence or absence of GFP expression [30,33]. Adapted with permission from Dydovik et al. [30] and Kanakov et al. [33].

Until now we have considered relatively simple classes of inputs of the type that can be separated by a single threshold and do not overlap. In these cases, the SGN merely classifies binary inputs that switch between the simple states such as being absent or present, or above or below a line. Biological reality, however, inevitably poses more complex situations. Classifying a more complex input, such as a concentration of a biological solute or signalling protein that falls within an upper and lower threshold, can also be addressed with SGN design (Figure 5A). Classification of a given two-input signature, for instance 10–20 nM of solute X and 600–800 nM solute Y, can be achieved with an *ab initio* designed SGN (Figure 5B) but begins to place a significant burden on the SGN designer (human or machine) to engineer or source sensor elements with the precise desired sensitivity to detect the two different solute concentration ranges. For example a given SGN design may require multiple promoters, each sensitive to different concentrations of the same, or different, solutes. In this situation, it is essential for the overall function of the network that there is no ‘cross-talk’ between the different inputs and the different promoters intended to be activated or repressed in response to those inputs. For instance, if solute A induces promoter A, but also induces promoters B, C and D unintentionally, the conditionality of outputs is compromised. As such, ‘orthogonal’ partners of inducer and promoter must be identified, in which a given inducer influences only a specific promoter type and has no effect on any other promoter. This orthogonality is a non-trivial objective for synthetic biologists [28] because it is arguably a defining feature of natural biology that genes within a genome tend to influence each other's expression [29].

### Ensembles of SGNs for classification of complex inputs

To meet these challenges, so-called ‘ensemble’ classifiers have been proposed [30,31]. The ensemble concept requires establishment of a heterogeneous population of simple classifier SGNs that encompasses a random distribution of sensitivities to input signals, each responding to only a narrow range of input levels. The overall output signal is the sum of the outputs of each SGN of the population and so can be considered as a tuneable collective response.

The SGN set out in Figure 5B features distinct ribosome binding site (RBS) elements, RBS_U1_ and RBS_U2_, which respond to a distinct concentration of their cognate input molecules, X_1_ and X_2_, respectively. High throughput (HTP) mutation approaches could be readily applied to generate a diverse library of RBS variants from RBS_U1_ to RBS_Un_. Once each variant has been introduced into cells, a population is generated harbouring an ensemble of SGNs with different input sensitivities. Across the ensemble population expression of the reporter would produce a bell-shaped response curve. The randomised sensitivity of the sensor RBS within each SGN of the ensemble is key. This distribution of sensitivities controls the position of the maximal signal output produced in response the concentration of a chemical input signal.

The ensemble of SGNs could be trained by selective deletion of the cells hosting SGNs that produce an incorrect response to positive or negative control signals. Total ensemble size, in terms of cell numbers, can be maintained by addition of new cells or by proliferation of the remaining non-deleted cells. Furthermore, probabilistic deletion, whereby incorrectly responding cells would have a finite probability of persisting within the ensemble population, would enable the ‘soft learning’ required for classification of input signals that have regions of overlap.

**Figure 6:**
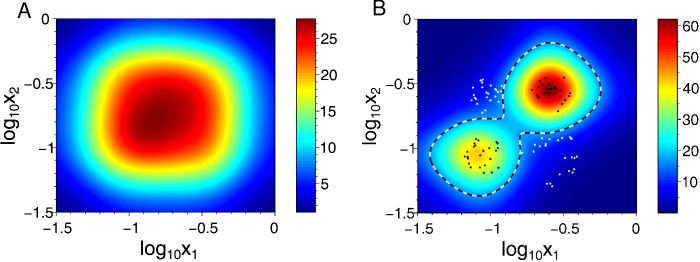
Simulation of an ensemble SGN soft learning how to classify overlapping input signals (**A**) The signals from two inputs, X_1_ and X_2_, overlap and therefore result in production of an overlapping output (red region) from an untrained ensemble SGN population. (**B**) After such a population has undergone loss of certain cells (indicated by white dots) due to selection pressure, mathematical modelling by Kanakov et al. [33] predicts that a classification border (within black and white dashed line) will emerge in respect to the output signal from the remaining cells (black dots). These remaining cells, and the ensemble of SGNs they harbour, can be considered as a ‘trained classifier’, which has undergone ‘soft learning’. The colour code of the heat map indicate relative change in response of the ensemble classifier, in arbitrary units. Adapted with permission from Kanakov et al. [33].

The sharp bell-shaped output of single synthetic circuits makes it possible to meet the challenge of distinguishing input classes that have a complex structure in the signal space. Effectively, training reshapes the distribution of individual sensitivities in the population, allowing them to cover the signal subspace corresponding to one of the classes by a union of ‘pixel’ responses. As a result, the SGN ensemble can be trained to classify inputs that are not linearly separable (Figure 6).

As SGN size and complexity increases so challenges in biological implementation also tend to increase, such as the availability of orthogonal genetic constructs. Excellent work by Nielsen et al. [32] demonstrated a robust system, Cello, for design and assembly of up to 45 SGNs with intended function. For ensemble SGNs (Figures 5 and 6), selection and deletion of variants can be performed to ensure cross-reaction of inputs does not dampen the collective response. As such in future the design space for large-scale SGNs may be limited only by the functional diversity of possible SGN component sequences and the metabolic capacity of a chosen cell type to replicate and express hosted SGNs.

## Intercellular communication between synthetic gene networks

Further sophistication in ensemble SGN design is likely to be achieved by integration with engineered intercellular communication. A study by Kanakov et al. [31] demonstrated that quorum sensing could be used to coordinate the function of designed genetic elements that have been distributed across different sub-groups of cells. They showed that toggle switch and oscillator functions could arise from these distributed, coordinated SGNs in a predictable and controllable manner. These distributed, coordinated SGNs were sensitive to modulation by external chemical signalling and the growth dynamics of the host cell population. This opens exciting possibilities for implementing dynamical decision making using distributed SGNs. Terrel et al. [34] also took a major step toward experimental implementation of distributed SGNs capable of classification. They demonstrated a system in which the presence of input was reported by a nanoparticle binding event that could occur only when two different cell types detected input signal.

## Applications and implications of bio-artificial intelligence

Synthetic biology has the potential to disruptively reconfigure goods and services that are today bio-based, such as vaccines, and those that are today mainly non-biological, such as sensor devices and computation [35]. The ‘designed’ biology envisioned by this approach will remain only a vision until basic research enables engineers to build and test sophisticated biological devices that perform predictably within parameters accurately described by mathematical modelling [35]. A major challenge for this vision is to build well-characterised, SGNs that go beyond discrete functions (sensing, oscillating) to incorporate the learning SGNs discussed here and networks of networks that provide new functions in cells and consortia of multiple cell types.

The steadily increasing number of advances in DNA synthesis and assembly make combinatorial assembly of large SGNs accessible and practical. Generating large libraries of DNA fragments of differing sequence allows selection of variants that function well and deletion of variants that perform poorly–in effect an evolutionary approach. Furthermore, modular assembly of large DNA molecules allows specific subsections to be removed and replaced with different variants, while the rest of the molecule is unchanged. Together these approaches mean evolutionary strategies can be used to find optimal solutions to circuit design, while modular approaches are used for debugging and error correction.

For example, multiple fragments composing a biosynthetic or signalling pathway can be assembled using a variety of methods for parallel ligation of multiple DNA fragments. Readers interested in detailed discussion of these DNA assembly methods should consult reports by Engler et al. [36] of the ‘MoClo’ method and by Weber et al. [37] of the ‘Golden Gate’ assembly method. Several methods have also been developed specifically for manipulation of very large (100 kilo base pairs and larger) DNA fragments [38–40]. Methods such as these have ultimately enabled assembly of entire bacterial and eukaryotic chromosomes [41,42].

Possible industrial applications of SGNs that can learn include designing cells that can respond to large, small, intended and unintended perturbations in bioprocess environments while maintaining optimal productivity, such as biotherapeutic production, resource utilisation or biosynthesis of high value chemicals. Smart cells that can respond to the physiological status of the patient in a sophisticated manner could also expand the application and robustness of whole cell therapeutic approaches.

Advances in conventional AI have raised concerns around the use of AI technologies in ways that would not be acceptable to wider society. Examples include the use of voice recognition in public spaces for surveillance purposes or deploying autonomous robots to work as counsellors, soldiers, carers or judges [43]. Bio-artificial intelligence (BI) could enable pheromone recognition or detection of a person's unique signature of volatile biological molecules. Of course these are purely long-term considerations, but we suggest it is prudent to monitor development in the field of AI as an indicator of the possible challenges BI might pose in future. A recent example of such precautionary oversight is the appointment of an ethics board at AI company Lucid (Austin, Texas, USA).

To date no reports exist of the application of SGNs in a commercial biomanufacturing process. As such, the current boundaries of synthetic biology must be pushed in order to deliver enhanced capabilities and a new era of ‘intelligent bio-manufacturing’. This might include deployment of ‘smart’ cells that can adapt to dynamic changes in their production (e.g. bioreactor) or application (e.g. organ, tissue) environments. As the global synthetic biology market grows, developing such capabilities will become a key challenge that will require the development of techniques across an increasingly broad palette of SGN architectures.

## Summary

- The classic Perceptron function for linear classification of inputs could in theory be implemented using ‘Teacher’ and ‘Student’ SGNs.
- SGNs have been designed to perform Pavlovian associative learning.
- Simulations in silico have provided preliminary confirmation that Pavlovian associative learning and Perceptron-based linear classification could be encoded in SGNs.
- SGNs and experimental schemes have been proposed that could be capable of evolving increased levels of diversity, enabling classification of complex input data.
- In future ‘bio-artificial intelligence’ may eventually pose ethical concerns that parallel those raised by recent developments in conventional artificial intelligence.

## Abbreviations

AI: artificial intelligence
BI: bio-artificial intelligence
BST: biological student–teacher
IPTG: isopropyl β-D-1-thiogalactopyranoside
PFNM: positive feedback/negative modified
RBS: ribosome binding site
SGN: synthetic gene network

## References

[1] Ghahramani Z. (2015). Probabilistic machine learning and artificial intelligence. Nature.

[2] Nakagaki T., Yamada H., Toth A. (2000). Maze-solving by an amoeboid organism. Nature.

[3] Tero A., Takagi S., Saigusa T., Ito K., Bebber D.P., Fricker M.D. (2010). Rules for biologically inspired adaptive network design. Science.

[4] Saigusa T., Tero A., Nakagaki T., Kuramoto Y. (2008). Amoebae anticipate periodic events. Phys. Rev. Lett..

[5] Mangan S., Alon U. (2003). Structure and function of the feed-forward loop network motif. Proc. Natl. Acad. Sci..

[6] Ma W., Trusina A., El-Samad H., Lim W.A., Tang C. (2009). Defining network topologies that can achieve biochemical adaptation. Cell.

[7] Jones B., Stekelo D., Rowe J., Fernando C. (2007). Is there a liquid state machine in the bacterium *Escherichia coli*?. Proceedings of IEEE Symposium on Artificial Life.

[8] Gandhi N., Ashkenasy G., Tannenbaum E. (2007). Associative learning in biochemical networks. J. Theor. Biol..

[9] Fernando C.T., Liekens A.M., Bingle L.E., Beck C., Lenser T., Stekel D.J. (2009). Molecular circuits for associative learning in single-celled organisms. J. R. Soc. Interface.

[10] Qian L., Winfree E., Bruck J. (2011). Neural network computation with DNA strand displacement cascades. Nature.

[11] Bates R., Blyuss O., Alsaedi A., Zaikin A. (2015). Effect of noise in intelligent cellular decision making. PLoS One.

[12] Borg Y., Ullner E., Alagha A., Alsaedi A., Nesbeth D.N., Zaikin A. (2014). Complex and unexpected dynamics in simple genetic regulatory networks. Int. J. Mod. Phys. B.

[13] Baştanlar Y., Ozuysal M. (2014). Introduction to machine learning. Methods Mol. Biol..

[14] Stormo G.D., Schneider T.D., Gold L., Ehrenfeucht A. (1982). Use of the ‘Perceptron’ algorithm to distinguish translational initiation sites in *E. coli*. Nucleic Acids Res..

[15] Rosen-Zvi M. (2000). On-line learning in the Ising perceptron. J. Phys. A: Math. Gen..

[16] Bernstein H.C., Carlson R.P. (2012). Microbial consortia engineering for cellular factories: *in vitro* to *in silico* systems. Comput. Struct. Biotechnol. J..

[17] Perry N., Nelson E.M., Timp G. (2016). Wiring together synthetic bacterial consortia to create a biological integrated circuit. ACS Synth. Biol..

[18] Suzuki N., Furusawa C., Kaneko K. (2011). Oscillatory protein expression dynamics endows stem cells with robust differentiation potential. PLoS One.

[19] Pavlov I.P. (1927). Conditioned Reflexes: An Investigation of the Physiological Activity of the Cerebral Cortex (translated by G.V. Anrep).

[20] Ginsburg S., Jablonka E. (2010). The evolution of associative learning: a factor in the Cambrian explosion. J. Theor. Biol..

[21] Gardner T.S., Cantor C.R., Collins J.J. (2000). Construction of a genetic toggle switch in *Escherichia coli*. Nature.

[22] Ajo-Franklin C.M., Drubin D.A., Eskin J.A., Gee E.P.S., Landgraf D., Phillips I. (2007). Rational design of memory in eukaryotic cells. Genes Dev..

[23] Lu T.K., Khalil A.S., Collins J.J. (2009). Next-generation synthetic gene networks. Nat. Biotechnol..

[24] Farzadfard F., Lu T.K. (2014). Genomically encoded analog memory with precise *in vivo* DNA writing in living cell populations. Science.

[25] Yang L., Nielsen A.A., Fernandez-Rodriguez J., McClune C.J., Laub M.T., Lu T.K. (2014). Permanent genetic memory with >1-byte capacity. Nat. Methods.

[26] Nishimura K., Fukagawa T., Takisawa H., Kakimoto T., Kanemaki M. (2009). An auxin-based degron system for the rapid depletion of proteins in nonplant cells. Nat. Methods.

[27] Giuraniuc C.V., MacPherson M., Saka Y. (2013). Gateway vectors for efficient artificial gene assembly *in vitro* and expression in yeast *Saccharomyces cerevisiae*. PLoS One.

[28] Rusk N. (2014). Orthogonal logic gates. Nat. Methods.

[29] Phillips P.C. (2008). Epistasis—the essential role of gene interactions in the structure and evolution of genetic systems. Nat. Rev. Genet..

[30] Didovyk A., Kanakov O.I., Ivanchenko M.V., Hasty J., Huerta R., Tsimring L. (2015). Distributed classifier based on genetically engineered bacterial cell cultures. ACS Synth. Biol..

[31] Kanakov O., Laptyeva T., Tsimring L., Ivanchenko M. (2016). Spatiotemporal dynamics of distributed synthetic genetic circuits. Phys. D.

[32] Nielsen A.A., Der B.S., Shin J., Vaidyanathan P., Paralanov V., Strychalski E.A. (2016). Genetic circuit design automation. Science.

[33] Kanakov O., Kotelnikov R., Alsaedi A., Tsimring L., Huerta R., Zaikin A. (2015). Multi-input distributed classifiers for synthetic genetic circuits. PLoS One.

[34] Terrell J.T., Wu H.-C., Tsao C.-Y., Barber N.B., Servinsky M.D., Payne G.F. (2015). Nano-guided cell networks as conveyors of molecular communication. Nat. Commun..

[35] Pais-Vieira M., Chiuffa G., Lebedev M., Yadav A., Nicolelis M.A. (2015). Building an organic computing device with multiple interconnected brains. Sci. Rep..

[36] Engler C., Gruetzner R., Kandzia R., Marillonnet S. (2009). Golden gate shuffling: a one-pot DNA shuffling method based on type IIs restriction enzymes. PLoS One.

[37] Weber E., Engler C., Gruetzner R., Werner S., Marillonnet S. (2011). A modular cloning system for standardized assembly of multigene constructs. PLoS One.

[38] Gibson D.G., Young L., Chuang R.Y., Venter J.C., Hutchison, 3rd C.A., Smith H.O. (2009). Enzymatic assembly of DNA molecules up to several hundred kilobases. Nat. Methods.

[39] Noskov V.N., Karas B.J., Young L., Chuang R.Y., Gibson D.G., Lin Y.C. (2012). Assembly of large, high G+C bacterial DNA fragments in yeast. ACS Synth. Biol..

[40] de Kok S., Stanton L.H., Slaby T., Durot M., Holmes V.F., Patel K.G. (2014). Rapid and reliable DNA assembly via ligase cycling reaction. ACS Synth. Biol..

[41] Annaluru N., Muller H., Mitchell L.A., Ramalingam S., Stracquadanio G., Richardson S.M. (2014). Total synthesis of a functional designer eukaryotic chromosome. Science.

[42] Hutchison C.A., Chuang R.Y., Noskov V.N., Assad-Garcia N., Deerinck T.J., Ellisman M.H. (2016). Design and synthesis of a minimal bacterial genome. Science.

[43] Russell S., Hauert S., Altman R., Veloso M. (2015). Robotics: ethics of artificial intelligence. Nature.

